# Supplementary material for: A Perspective for Ménière’s Disease: In Silico Investigations of Dexamethasone as a Direct Modulator of AQP2
Source: Biomolecules. 2022 Mar 28;12(4):511. doi: 10.3390/biom12040511 (PMC9028334; doi:10.3390/biom12040511)
Supplement: Supplementary file 1 [file biomolecules-12-00511-s001.zip › Mom-et-al-2022-Dex-AQP2-supplementary.pdf]

Supplementary

pf calculation and correction with the Dk constant

The single-channel permeabilities (*pf*) were computed independently for each protomer based on the collective diffusion model proposed by Zhu et al[1]. Accordingly *pf* was computed from the slope of the mean-square displacement (MSD) of the collective water coordinate. The slope was computed by fitting a line to the MSD up to a time displacement of 100 ps. For each protomer, the analysis was carried out on water molecules within a cylinder of length 8 angströms, centered on the two NPA motifs asparagines Cα center of geometry, that is within the narrowest part of the channel where water molecules are known to form a single file continuum.

In a previous work[2], we introduced a correction constant to accentuate the effect of the ar/R constriction calculated from the free energy profiles as follows:

$$Dk = [\ 2E_0 - E_{arR}\ ] / E_0$$

with *Dk* the unit free correction constant ; E_{arR} the free-energy at the ar/R constriction site and E_0 the free energy corresponding to the highest free-energy barrier in the channel section used to calculate *pf*. E_0 must be smaller than E_{arR} for the correction to be applied. *Dk* integrates the contribution of the ar/R constriction to water diffusion and is comprised between 1 and 0 : when the difference between the two free-energy barriers tends toward 0, *Dk* tends toward 1. On the other hand, the higher the free-energy barrier of the constriction is, the smaller *Dk* is, eventually reaching a limit of the correction when E_{arR} becomes more than twice as high as E_0. In this case, *Dk* becomes negative and is considered as equal to 0. To adjust the *pf*, one has to multiply it by *Dk*:

$$pf_{corrected} = pf \times Dk$$

In the present study, we broaden this *Dk* correction to other free energy barrier than the ar/R constriction free energy barrier only. Hence, *E0* still corresponds to the highest free-energy barrier included in the channel section used to calculate pf. And E_{arR} is replaced by E_{HB} (*HB* standing for Highest Barrier) and designate the highest free energy barrier outside of the channel section used to calculate *pf*:

$$Dk = [\ 2E_0 - E_{HB}\ ] / E_0$$

This approach was used to compute corrected pf values of figure 5 and is detailed on supplementary figures1.

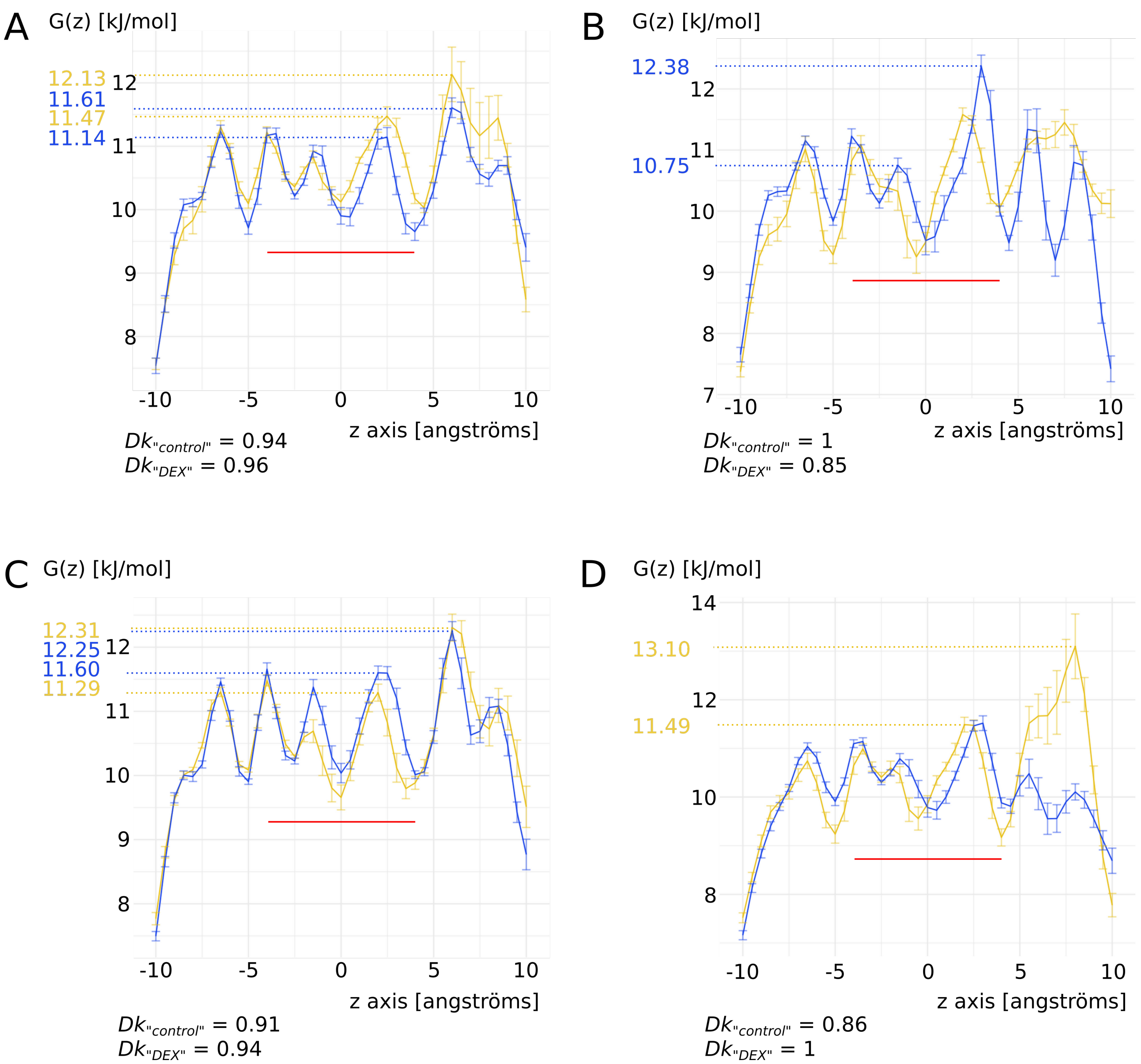

Supplementary figureS1. Dk constant for pf correction. *For each chain (**A**, **B**, **C** and **D**) is displayed free energy profiles of water zoomed on the 20 Å of the water channels. The z axis coordinates are relative to the center of geometry of the Cα of the two asparagines of the NPA motifs (i.e. the center of the conducting pore). The two conditions of the second experimental setup “control” (in yellow) and “DEX” (in blue) are compared. The red line indicates the region used for pf calculation. The free energy values used as E_0 and E_{HB} for Dk constant calculation are indicated by dashed lines.*

The energetic barrier does not always correlate with the steric barrier

Figure S2 illustrates the absence of correlation between water counts and size of the ar/R constriction between the four chains in the third experimental setup (see methods). The conformational and charge repartition changes induced by sodium interaction have a clear impact on the size of the ar/R constriction through their impact on arginine 187 side chain position inside the lumen of the pore of chain A. However, the modulatory effect of dexamethasone binding on chain B and chain C is more subtle and impacts water flux through modifying the pore electrostatic profile rather than by simple steric occlusion (see results). More experiments need to be performed to shed more light on the molecular mechanisms involved.

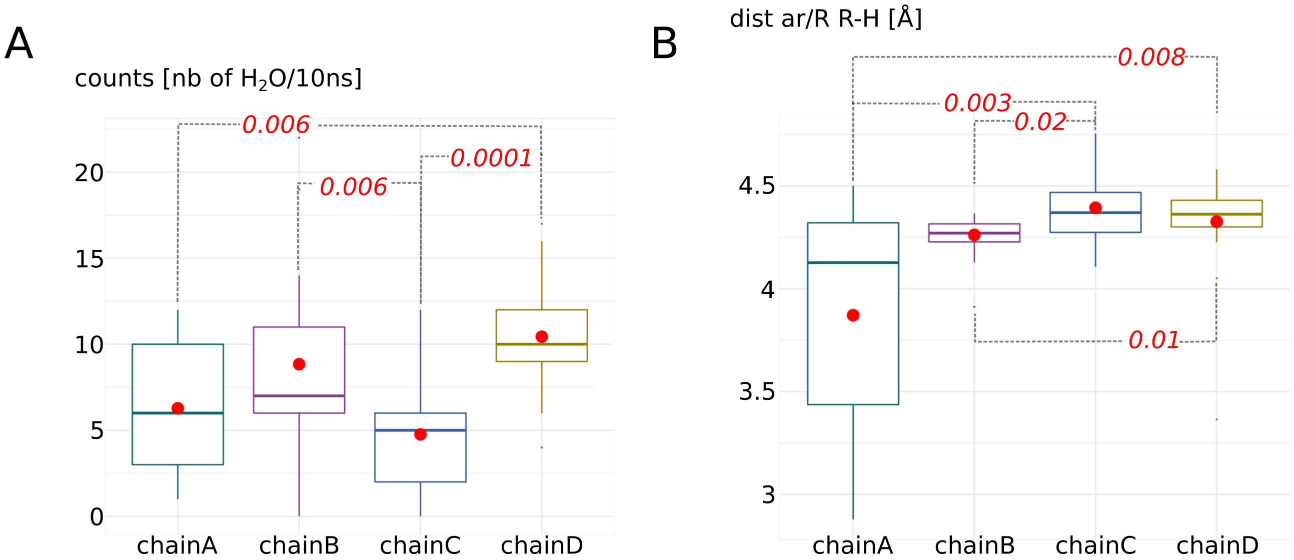

Supplementary figures2.Water channels permeabilities do not correlate with minimal ar/R R-H distances in third experimental setup. A. *Statistical analysis of the water counts (number of water molecules crossing the 30 Å long transmembrane channel per 10ns sub-section of the trajectory). Non parametric Wilcoxon test with post hoc Bonferroni correction is used to compare the four chains. Significant differences are indicated by dashed lines with the corresponding p. value in red italic.* ***B.*** *Statistical analysis of the minimal distance between the arginine and the facing histidine of the ar/R constriction. Non parametric Wilcoxon test with post hoc Bonferroni correction is used to compare the four chains. Significant differences are indicated by dashed lines with the corresponding p. value in red italic.*

Dexamethasone stabilization in POPC bilayer

In the last experimental setup, we observed spontaneous stabilization of dexamethasone inside the POPC bilayer (figure S3). For both compartment, the dexamethasone was stabilized inside the upper membrane (relatively to the box coordinates). This is in favor of our hypothesis stipulating that AQP2 dipole moment orients dexamethasone molecules inside the simulation box. If true, this would result in the dexamethasone molecules presenting their negative pole toward the upper membrane where the positively charged choline groups of POPC would constitute good interacting partners. On the opposite, no stabilization event of dexamethasone into the lower membranes occurred during the 250 ns of simulation. However these preliminary data are not sufficient to conclude.

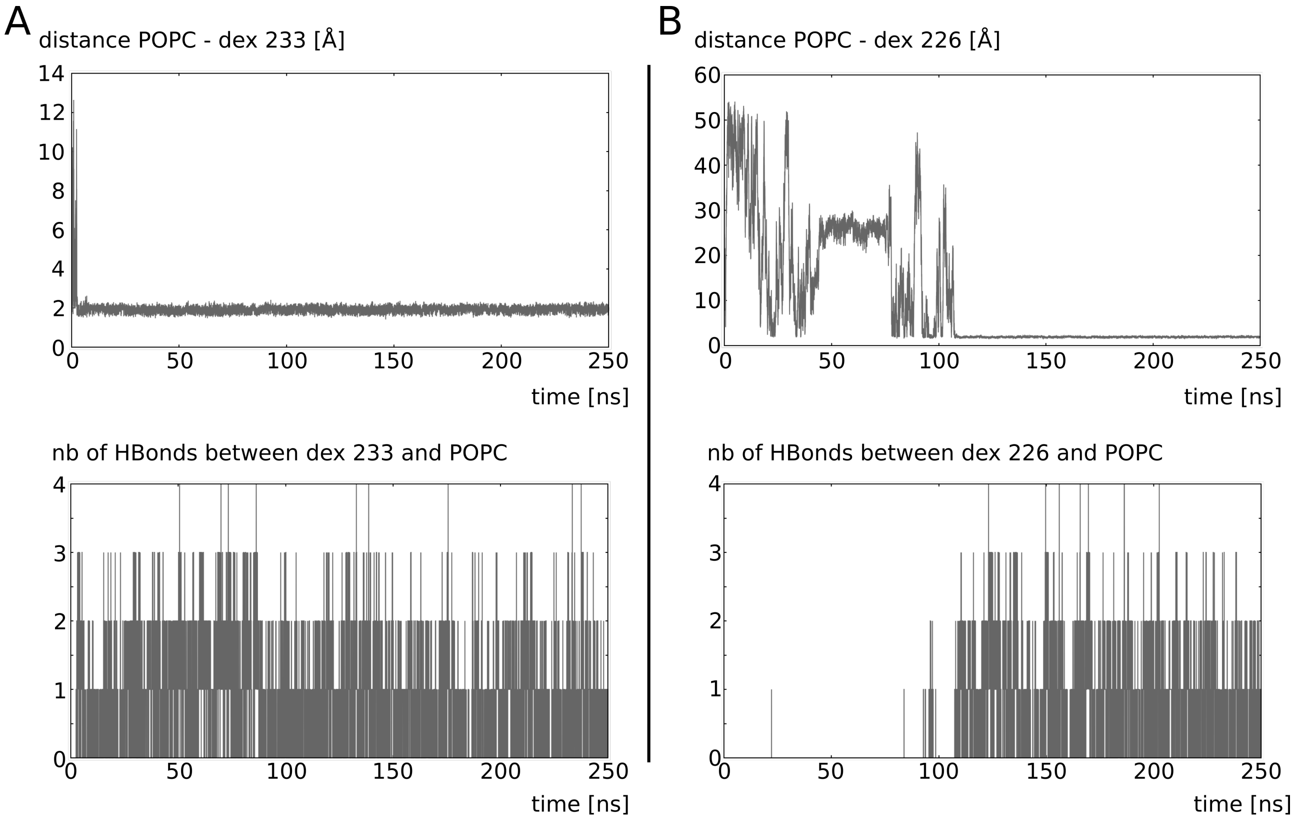

Supplementary figureS3. Dexamethasone stabilization in POPC bilayer. A. *Dexamethasone residue 233 of intra-cellular compartment and* ***B.*** *Dexamethasone residue 226 of extra-cellular compartment. For both molecules, the minimal distance between the dexamethasone and the POPC is displayed as a function of time as well as the number of hydrogen bonds established between them as a function of time. The data are extracted from the third experimental setup (see methods).*
